# Choroidopathy and kidney disease: a case report and review of the literature

**DOI:** 10.1186/1757-1626-2-7425

**Published:** 2009-05-12

**Authors:** Nirav V Kamdar, Amsalu Erko, Jason S Ehrlich, Jonathan W Kim, Neeraja Kambham, Glenn M Chertow

**Affiliations:** 1 Division of Nephrology, Departments of Medicine, Stanford University School of MedicinePalo Alto, CAUSA; 2 Ophthalmology, Stanford University School of MedicinePalo Alto, CAUSA; 3 Pathology, Stanford University School of MedicinePalo Alto, CAUSA

## Abstract

The patient was a 41 year-old Mexican American women who presented with a decrease in visual acuity along with periorbital and peripheral edema. She was diagnosed with bilateral serous retinal detachment and diffuse proliferative lupus nephritis. She improved considerably in hospital after treatment with corticosteroids.

## Case presentation

A 41 year-old Mexican American woman was transferred from a community hospital emergency department to the Ophthalmology service of a tertiary care academic medical center due to progressive, bilateral decrease in visual acuity over the 2-3 weeks prior to admission. She was in her usual state of health until two months before admission when she observed pedal followed by periorbital edema, which increased in severity and was followed by darkened urine. Her past medical history was significant for Graves' disease diagnosed six months earlier and treated with brief courses of methimazole and oral prednisone. Methimizole was discontinued by her primary care provider due to myositis. Prednisone was self-discontinued by the patient due to concerns regarding the long-term complications of steroid use.

When the patient's vision acutely decreased to the point where she was unable to drive or work, she sought medical care in her community. She was noted to have spectacle-corrected visual acuity in the range of 20/200 to 20/400 in both eyes, as well as marked hypertension (192/115 mmHg). She was treated with labetalol. A computed tomography (CT) scan was performed that demonstrated preseptal eyelid edema and mild proptosis of both eyes. She was transferred with a presumptive diagnosis of Grave's orbitopathy requiring urgent orbital decompression by an oculoplastics specialist. A dose of intravenous methylprednisolone (1000 mg) was administered en route.

**Figure 1.**
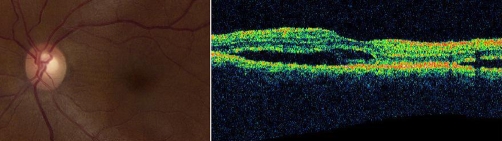
Fundus photograph of the left eye showing macular striae (left panel) and optical cohrerence tomography scan through the left macula with collections of subretinal fluid (right panel). The photographs were taken on hospital day 4 after several days of intravenous steroid therapy, at which time the patient’s vision had already significantly improved.

Upon arrival, a complete ophthalmologic examination was performed. The patient noted that in the hours following initial administration of intravenous prednisolone, her vision had improved. On our examination, best-corrected visual acuity was 20/100 in each eye. Pupils were round, reactive, and symmetric with no afferent pupillary defect. Extraocular movements were full and symmetric; there was no diplopia in any field of gaze and no evidence of eyelid retraction. Anterior segment examination revealed 2+ spongy periorbital edema, 1-2+ white conjunctival chemosis with no conjunctival injection, and minimal proptosis clinically. The remainder of the anterior segment was normal in both eyes. Dilated fundusopic examination revealed subtle striae in the macula of both eyes (Figure 1), as well as shallow, non-rhegmetogenous retinal detachments inferiorly in both eyes with shifting subretinal fluid. These findings were not consistent with Graves' orbitopathy. A repeat CT scan demonstrated mild bilateral orbital proptosis, minimal enlargement of the extraocular muscles and no crowding of the optic nerves at the orbital apex.

Physical examination was also notable for hypertension, bilateral facial rash, pedal edema and abdominal ascites. Sensorineural hearing was grossly intact by Weber and Rinne's tests. The scalp showed thinned, sparse hair that she affirmed was unchanged since adolescence.

On review of systems, she had diarrhea for the two weeks before her admission and mild hearing loss. She also noted myopathy and pedal paresthesias. She had a 15 pack-year history of tobacco use. Family history was positive for diabetes mellitus and osteoporosis, but no kidney, thyroid, or autoimmune disease.

Since the diagnosis of Graves' orbitopathy appeared in doubt, additional diagnostic tests were performed. Laboratory studies were notable for an elevated leukocyte count of 16.0 K/microliter, and a microcytic anemia with an hematocrit of 32.7% and a MCV of 79.4 fL. Thyroid panel revealed normal T3, free T3, and free T4, with a suppressed TSH (0.03 mIU/L) and positive thyroid autoantibodies with a TSI of 2.2 (normal <0.3). Her electrolytes were normal, but the blood urea nitrogen (BUN) and serum creatinine were 46 mg/dL and 2.0 mg/dL, respectively. There had been no prior evidence of impaired kidney function. The Nephrology service was consulted.

Urinalysis demonstrated proteinuria and hematuria with a spot protein/creatinine ratio of 7.58. The total serum cholesterol concentration was 469 mg/dL. Serum albumin was markedly reduced at 1.3 g/dL. Hepatitis B and C antibodies were negative. Serum complements were low (C3 and C4 concentrations 26 and 2.3 mg/dL, respectively). Antinuclear and anti-double stranded DNA antibodies were positive. Anti-glomerular basement membrane (GBM) antibodies and anti-nuclear cytoplasmic antibody (ANCA) studies were negative.

**Figure 2.**
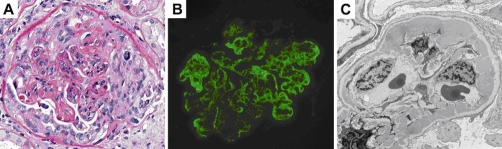
Light microscopy, immunoflorescence and electron microscopy of kidney biopsy section taken on hospital day 3 (see text).

On day three of her hospitalization, the patient underwent a percutaneous kidney biopsy without complication. The biopsy demonstrated World Health Organization (WHO) class IV (diffuse proliferative) lupus nephritis with endocapillary proliferation, wire loops, cellular crescents, and karyorrhectic debris. Although the biopsy showed largely active immune processes, there was evidence of chronic disease with fibrocellular crescents and segmental membranoproliferative features with tubular atrophy and interstitial fibrosis. Electron microscopy demonstrated hypercellular glomeruli with mesangial and endocapillary proliferation with infiltrating lymphocytes, neutrophils, and macrophages. There were abundant electron dense deposits in the mesangium and subendothelium with immunoflorescence evidence of IgG, C3, IgA, IgM, and C1q (Figure 2). The patient continued on intravenous corticosteroids. Her best-corrected vision continued to improve, to 20/40 in both eyes on hospital day four.

## Discussion

Ophthalmologic complications of kidney disease in general and of systemic lupus erythematosus (SLE) in particular are not uncommon but are rarely threshold signs for nephrologists to establish the diagnosis of acute or chronic kidney disease. Despite their vastly different functions, both organs are vulnerable to similar pathological processes. One can broadly categorize these complications into genetic, vascular/volume-related, inflammatory, and systemic/metabolic disease-related etiologies.

Several genetic diseases have both renal and ophthalmologic manifestations. Alport's syndrome, an X-linked recessive and autosomal recessive disease, has sensory degredations including posterior corneal dystrophy, juvenile arcus and retinal pigmentation changes in addition to hematuria. Cystinosis largely plagues neonates with kidney failure and corneal dysfunction. Fabry's disease, a lysosomal storage disease, presents with kidney failure and corneal vessel tortuosity and anterior subcapsular opacities in infants. Oculocerebrorenal syndrome (Lowe's syndrome), is characterized by renal tubular acidosis in conjunction with corneal keloids, glaucoma, and cataracts.

Fluid dynamics play a joint pathophysiological role in impaired kidney function and associated ophthalmologic disease, as the interstitial spaces of the eye are subject to Starling's principles of fluid dynamics. Fluid will tend to shift toward the interstitial space if hydrostatic pressure increases, oncotic pressure decreases (due to reduced plasma proteins) or if capillary vessels become more permeable to fluid movement. These conditions tend to be particularly problematic in the setting of glomerular disease. Older primate experiments demonstrated that with highly elevated blood pressures, one would observe bilateral serous retinal detachments [1]. Notably, any process that alters the integrity of the glomerular basement membrane can lead to proteinuria, reduced plasma protein concentration, and thus a decrease in plasma oncotic pressure which leads to fluid displacement into interstitial spaces such as compartments adjacent to the retina of the eye.

Several systemic diseases contribute to both ocular and renal pathology. The most notable are the long-term sequelae of diabetes mellitus in which one sees kidney damage characterized initially by microalbuminuria followed by glomerulosclerosis and ultimately kidney failure; in the eye, hyperglycemia causes increased glucose to enter retinal cells and become converted to sorbitol. The excess sorbitol concentration increases the osmolality within retinal cells and results in cell swelling, which ultimately leads to retinal cell dysfunction. Both organ systems are also highly susceptible to immune complex deposition and damage that leads to fluid displacements into the interstitium. As evidence, experimentally induced type III hypersensitivity reactions in rabbits resulted in bilateral uveitis and glomerulonephritis in the same animals [2,3]. Many of the glomerulonephritidies involve systemic immune-related responses with B-lymphocyte hyperactivity or autoantibody production. These reactions are best illustrated in the systemic vasculitidies such as polyarteritis nodosa and Wegener's granulomatosis. In the latter, antibodies against type-IV collagen produce both hematuria and ocular inflammatory reactions that result in keratitis, scleritis, and episcleritis.

Systemic lupus erythematosus (SLE) is a chronic systemic autoimmune disease whose pathology affects multiple organ systems including the eye. Typically, the disease involves multiple organs including cutaneous, rheumatologic, renal, cardiac, and neural systems. The diagnosis can be made if four of eleven diagnostic criteria are met from the American College of Rheumatology [4]. Kidney disease complicates approximately 50% of documented SLE; characteristic findings include microscopic hematuria, proteinuria (often above 3 g/day), hypertension and impaired kidney function, depending on the severity, duration and subtype of the disease. The ocular process most commonly associated with SLE is keratoconjunctivitis sicca [5], a dry eye syndrome resulting from damage in the lacrimal secretory glands. A more concerning ocular finding is scleritis, an inflammation of the sclera that manifests as intraocular inflammation and choroidal thickening. Retinal involvement is also common with the classic findings of cotton-wool spots as well as retinal vasculitis. Our patient was particularly interesting because these typical ophthalmic findings were absent, most notably the vasculitis and evidence of intraocular inflammation. Choroidal involvement is much less common but has been reported to present with bilateral serous retinal detachment [5]. The onset of ophthalmic manifestations of lupus may herald a flare of systemic disease and should prompt appropriate investigation [6].

The presentation of choroidopathy with SLE follows some typical epidemiological patterns of SLE; for example, 93% of patients who presented with choroidopathy and retinal detachments with SLE occurred in women [7]. Nguyen and colleagues [7] documented that between 1968 and 1998, 96% of patients were known to have a diagnosis of SLE at the onset of choroidopathy. The exceptions occurred only in patients who did not have primary and long-term access to healthcare; patients then presented to emergency departments with blurred vision and lupus was subsequently diagnosed during the basic work-up, as occurred here. Our patient was an undocumented resident without access to primary care except at a local free clinic near her home. Thus, the stimulus to seek medical care was her decreased visual acuity, despite observing unusual facial and pedal edema related to her SLE prior to visual difficulties.

Several groups who have observed bilateral retinal detachments in the setting of SLE have noted abnormalities of the retinal pigment epithelium (RPE) [7,8-10]. Matsuo and colleagues [9] hypothesized that anti-retinal pigment epithelium antibodies were involved in causing a dysfunction of the RPE which ultimately led to the development of serous retinal detachment. Other groups have observed immune complex deposition in the choroid with mononuclear infiltrates, whose cytokines might cause inflammation and fluid collections behind the retina [11]. Other theories have implicated systemic hypertension typically seen with SLE. Hypertension can cause choroidal vascular occlusions that would exacerbate edema within the retina [12]. Our patient demonstrated newly diagnosed hypertension related to her underlying kidney disease. Hypertension, in conjunction with severe hypoalbuminemia, may have contributed to the fluid accumulation and serous retinal detachments. It is noteworthy that we saw no evidence of retinal vasculitis on fluorescein angiography; this test only demonstrated the presence of subretinal fluid, but no findings consistent with vasculitis or choroidopathy.

Our patient was referred for presumed ophthalmological complications of her Graves' disease. Upon review and examination, it became clear that the reason for reduced visual acuity was retinal detachment. She had received high dose corticosteroids for presumptive treatment of Graves' ophthalmopathy. The effect of methylprednisolone on SLE, including the evolving nephritis, may have helped preserve her vision over the time of her hospital-to-hospital transfer. Upon discharge from the hospital, the patient's vision had improved. Interestingly, Hannouche and colleagues [8] report resolution of the choroidopathy after plasmapharesis, which indicates the importance of immune complex deposition in the pathophysiology of the disease as well as its management strategy, at least in some cases.

## Conclusion

Here we provide another important example of where ocular sequelae enlighten the diagnosis and management of kidney disease. Nephrologists should carefully attend to the eyes of patients with previous diagnosed SLE who present to the office or hospital with a flare of the systemic disease. More generally, choroidopathy should be recognized among the oculorenal syndromes, and considered in the setting of impaired vision or eye pain associated with glomerular disease.

## List of abbreviations

ANCA: Anti-nuclear cytoplasmic antibody
BUN: Blood urea nitrogen
CT: Computer tomography
DNA: Deoxyribonucleic acid
GBM: Anti-glomerular basement membrane
RPE: Retinal pigment epithelium
SLE: Systemic lupus erythematosus
TSH: Thyroid-stimulating hormone
